# Study on Moisturizing Effect of *Dendrobium officinale*, *Sparassis crispa*, and Their Compound Extracts

**DOI:** 10.1111/jocd.70189

**Published:** 2025-04-18

**Authors:** Hankun Ren, Peina Zha, Yueheng Liu, Weihong Zhang, Hong Meng, Taiju Di

**Affiliations:** ^1^ Beijing Academy of TCM Beauty Supplements Co. Ltd Beijing People's Republic of China; ^2^ Beijing Technology and Business University Beijing People's Republic of China

**Keywords:** aquaporin 3, Claudin‐1, *Dendrobium officinale*, filaggrin, hyaluronic acid synthetase, moisturizing, plants, *Sparassis crispa*

## Abstract

**Background:**

Recently, natural plants have been widely developed and applied in moisturizing cosmetics. *Dendrobium officinale* Kimura et Migo ( *D. officinale* ) is known as one of the “Nine Immortals” of Chinese herbal medicine, whereas *Sparassis crispa* (Wulf.) Fr. ( *S. crispa* ) is known as the “king of mushrooms”; both of which have excellent biological activity.

**Aims:**

To explore the effects of *D. officinale* polysaccharide (DOP) with different molecular weights and *S. crispa* on the expression of moisturizing‐related genes and verify the moisturizing performance of their complex.

**Methods:**

PCR was carried out to explore the aquaporin 3 (AQP3), hyaluronic acid synthetase1 (HAS1), HAS2, and HAS3 genes expression. Immunofluorescence (IF) analysis was used to test the protein level expression of hyaluronic acid (HA), AQP3, claudin‐1, and filaggrin (FLG) influenced by moisturizing composition in a reconstructed epidermis skin model. The ability of samples to resist cell drying damage was evaluated by a cell drying damage model. Furthermore, this study validated the effect of the compositions during their application in cosmetics through tests of skin moisture content, crow's feet, and skin elasticity.

**Results:**

The results showed that DOP with molecular weights of 100 k–500 kDa (Dalton) had higher effects on AQP3 gene expression compared to that with molecular weights of 10 k–100 kDa and 1 k–10 kDa. Additionally, the extract of *S. crispa* significantly promoted the expression of HAS1, HAS2, and HAS3 genes, which are genes encoding hyaluronic acid synthesis. In addition, the mRNA and protein expression levels of HA, AQP3, claudin‐1, and FLG were significantly increased as a result of the moisturizing composition consisting of DOP (100 k–500 kDa) and *S. crispa* . The application of the moisturizing composition markedly increased the skin moisture content, improved eye wrinkles, and enhanced skin elasticity.

**Conclusions:**

In summary, our study proved that *D. officinale* and *S. crispa* had good moisturizing effects, and as natural plant humectants, they may have broad applications in future moisturizing cosmetics.

## 1 Introduction

Moisturizing is a fundamental function of skin care cosmetics and an important factor in maintaining skin health and delaying skin aging [1]. Traditional humectants mainly include hygroscopic sealants that are represented by glycerol and mineral oil, and exogenous bionic ingredients such as sodium hyaluronate and ceramide. With the development of moisturizing products, the moisturizing effect of skincare products has also been found to enhance the endogenous moisturizing function of skin by regulating the expression of skin moisturizing genes and proteins and improving the skin's own state [2]. Multiple mechanisms such as the keratinocyte tight junction pathway, aquaporin pathway, and hyaluronic acid pathway are involved in skin moisturization [3, 4].

The recent pursuit of natural plant skincare products by consumers has prompted domestic and foreign cosmetics companies to pay more attention to the research and development of cosmetic plant ingredients. Polysaccharide, which is one of the important active ingredients in plants, has attracted extensive attention in recent years for its excellent moisturizing properties and has been applied in cosmetics as a moisturizing agent [5, 6].

Traditional Chinese medicine believes that problems such as dry skin are mostly due to yin deficiency of the liver and kidney, dryness and heat hurting body fluids, and loss of nourishment of the lung and spleen, which leads to a lack of nourishment of the skin [7, 8]. *Dendrobium officinale* Kimura et Migo ( *D. officinale* ), belonging to the *Dendrobium* of the *Orchidaceae genus*, is known as one of the “Nine Immortals” of Chinese herbal medicine [9]. It has the effects of nourishing Yin and clearing heat, nourishing the stomach and promoting fluid, moistening the lung and relieving cough, and can take into account the disadvantages of insufficient fluid in the spleen and stomach [10].

*D. officinale* has a wide range of pharmacological applications, which is due to it containing a variety of chemical substances, including polysaccharides, stilbenoids and their derivatives, lignans, coumarin, flavone, cinnamate, sesquiterpene, fatty acid, and alkaloids [11, 12]. Studies have shown that *D. officinale* is rich in polysaccharides and has skin care effects such as anti‐oxidation, anti‐aging, moisturizing, and anti‐inflammatory [13]. According to Cha et al. [14] and Li et al. [15], *D. officinale* polysaccharide (DOP) extract can increase the expression of FLG, caspase‐14, tight junction proteins (occludin and claudin1) and AQP3 gene to promote skin hydration. Therefore, *D. officinale* can promote the expression of skin moisturizing genes.

A further study on polysaccharides has also found that various biological activities of polysaccharides are closely related to their chemical characteristics, including monosaccharide compositions, molecular weights, chain conformations, types of glycosidic linkages, uronic acid contents, and structural modifications.

Research on conformation relationship of polysaccharides is mainly conducted to study the type of glycosidic bond, the conformation of main branch chain, the composition of monosaccharides, the three‐dimensional conformation, the type and number of groups, and the molecular weight of polysaccharides [16, 17]. For example, Wang et al. [18] have shown that the antioxidant activity of polysaccharides is related to their structural properties, including monosaccharide composition, molecular weight, and types of glycosidic linkages. Therefore, it is necessary to study the relationship between polysaccharide function and structure. However, thus far, there are only a few reports on the effects of *D. officinale* with different polysaccharide molecular weights on moisture‐related genes.

Mushroom has also been widely used in cosmetics in recent years. *Sparassis crispa* (Wulf.) Fr. ( *S. crispa* ) is known as the “king of mushrooms” that is rich in protein, amino acid, vitamin and other active ingredients and possesses different biological activities such as anti‐tumor, anti‐oxidation and immune regulation [19, 20]. *S. crispa* contains highly active biological and pharmacological ingredients (e.g., β‐glucan, anti‐fungal compounds (sparassol, methyl‐2,4‐dihydroxy‐6‐methylbenzoate, and methyldihydroxymethoxy‐methylbenzoate), ergosterol peroxides, and benzoate derivatives) that are useful in the treatment of human disease [21]. Among them, the content of β‐glucan in *S. crispa* is more than 40%, which is the highest among all fungi [22]. It has been reported that *S. crispa* can improve the turnover of stratum corneum and the content of dermal soluble collagen in collagen synthetic activity‐reduced model rats, in addition to reducing the skin water loss [22]. Nonetheless, there are only a few studies that investigate the moisturizing mechanism of *S. crispa* and the regulation of moisturizing factors.

Therefore, based on the concept of endogenous promotion of moisture gene expression, this study explored the effects of DOP with different molecular weights and *S. crispa* on the expression of moisturizing‐related genes. The study also verified the moisturizing performance of their complex. This research is expected to provide a theoretical basis for the application of moisturizing plants as raw materials in cosmetics.

## 2 Materials and Methods

### 2.1 Materials and Instruments

Materials used include *D. officinale* (Kunming Institute of Botany, Chinese Academy of Sciences), *S. crispa* (Yunnan Yujunlong Trading Co. Ltd.), human keratinocyte cell line HaCaT (Chinese Academy of Medical Sciences, Beijing, China), 3D epidermal skin model (Guangdong Biocell Bio Co. Ltd.), RNAiso Plus, reverse transcription kit, fluorescent dye (Accurate Bio), AQP3, Claudin‐1, FLG, HA antibody (Abcam), and paraformaldehyde (Biosharp).

Instruments used in the experiments include a CO_2_ incubator (Themo, 150I), an averted microscope (Olympus, CKX41), a real‐time PCR instrument (BioRad, CFX‐96), a laser confocal microscope (Leica, DM2500), HPLC (Agilent 1200), 7890B‐7000D GC–MS (Agilent Technologies Inc. CA, UAS), a skin moisture content tester (Corneometer CM 825, Courage and Khazaka), EvaSKIN (EOTECH), and a skin elasticity tester Cutometer (MPA580, Courage and Khazaka).

### 2.2 Sample Preparation

#### 2.2.1 Preparation of Polysaccharides From *D. officinale*

Dried stems of *D. officinale* were ground into a fine powder, mixed with water at a mass ratio of 1:60, stirred at room temperature for 20–30 min, and then extracted at 55°C–60°C for 1.5–2 h. *D. officinale* with molecular weights of 1 k–10 kDa, 10 k–100 kDa, and 100 k–500 kDa was collected and then concentrated at 70°C–75°C and 0–0.01 MPa to a solid content of 10%–15%. The solution was precipitated with 80% ethanol with a volume four times its volume and then recovered by filtration [14]. The sediment was dispersed in water at a ratio of 1:60.

#### 2.2.2 Preparation of *S. crispa* Extract

*S. crispa* was crushed and treated at 110°C and 0.19 MPa for 20 min. After that, it was mixed with water at a mass ratio of 1:60, stirred at room temperature for 30 min, and then extracted at 55°C–60°C for 2 h. The extract of *S. crispa* was collected as filtrate (treatment group, SCE(T)). To prepare *S. crispa* extract (untreated group, SCE(UT)), the sample was subjected to the same processes but without high temperature and micro pressure treatment.

#### 2.2.3 Preparation of Moisturizing Complex

In the experiment, we prepared different proportions of DOP and *S. crispa* . By comparing the moisturizing effect and skin feeling, we finally confirmed that the ratio of DOP and *S. crispa* was 2:1. Moisturizing complex (MC) was prepared by mixing DOP and *S. crispa* at a ratio of 2:1. DOP with molecular weights of 100 k–500 kDa prepared according to the method described in 2.2.1 was added with water at a mass ratio of 1:120. The extract was mixed with *S. crispa* (after high‐temperature and micro‐pressure treatment), and the mixture was stirred at room temperature for 30 min, extracted at 60°C for 2 h, and then filtered. The filtrate was collected.

### 2.3 Polysaccharide Tests

#### 2.3.1 Polysaccharide Content Test

The polysaccharide content in 100 k–500 kDa DOP after separation and purification was determined by the phenol‐sulfuric acid method.

#### 2.3.2 Monosaccharide Composition Test

The monosaccharide composition of DOP was examined by comparing its retention time with that of standard monosaccharides (fucose (Fuc), rhamnose (Rha), arabinose (Ara), galactose (Gal), glucose (Glc), xylose (Xyl), mannose (Man), ribose (Rib), Galacturonic acid (GalA), glucuronic acid (GlcA), mannuronic acid (ManA), and guluronic acid (GulA)). The 100 k–500 kDa DOP prepared in 2.2.1 was freeze‐dried, and its monosaccharide composition was determined by high‐performance liquid chromatography (HPLC). DOP (5 mg) was hydrolyzed with 4 M trifluoroacetic acid (TFA, 2 mL) at 110°C for 5 h. It was then dried under vacuum and derivatized with 0.05 mL of 1‐phenyl‐3‐methyl‐5‐pyrazolone (PMP) solution (0.5 M in methanol) and 0.05 mL of 0.3 M NaOH at 70°C for 60 min. Subsequently, 0.05 mL of HCl (0.3 M) was added for neutralization and 1.5 mL of chloroform was added for extraction; this step was repeated three times [23].

The data acquisition system was set up as follows: instrument, an Agilent 1200; detector type, UV detector; chromatographic column, C18 Agilent 4.6 mm × 250 mm × 5 μm; mobile phase A, 0.1 M KH_2_PO_4_ (PH6.8); mobile phase B: acetonitrile; mobile phase gradient, A:B = 82:18 (equal elution); flow rate, 1.0 mL/min; column temperature, 25°C; injection volume, 10 μL; and detection wavelength, 254 nm.

#### 2.3.3 Polysaccharide Bonding Structure

Freeze‐dried 100 k–500 kDa DOP sample prepared in 2.2.1 was dissolved in 500 μL of DMSO. The sample was added with 10 mg of NaOH and then incubated for 30 min before being added with 0.1 mL of CH3I and incubated for another 30 min. The temperature of a water bath was controlled at 18°C–20°C during incubation. The reaction was terminated with 1.0 mL of Na_2_S_2_O_3_ (4 mmol/L) and then extracted four times with 2.0 mL of CH_2_Cl_2_; after that, it was blow‐dried with a nitrogen stream. Then, the sample was hydrolyzed with TFA (2.0 M) and then reduced with 0.5 mL of NaBD_4_ (1.0 M). Acetic acid (20 μL) was added to terminate the reaction, followed by acetic anhydride to acetylate the sample. Finally, after being extracted with CH_2_Cl_2_, the sample was analyzed by GC–MS (Agilent 7890B; Agilent Technologies, USA) [23].

The injection volume was 1 μL at a split ratio of 10:1. High purity helium was used as a carrier gas. The initial temperature of 50°C was maintained for 1.0 min before being increased to 215°C at a rate of 40°C/min and maintained for 45 min. The mass spectrometric system (Agilent 7000d; Agilent Technologies, USA) was equipped with an electron bombardment ion source (EI) and a MassHunter workstation. The analyte was detected in a full SCAN mode at the mass scanning range of 30–600 (*m*/*z*).

### 2.4 Gene Expression Analysis

Cells were inoculated in 6‐well plates at a density of 2.5 × 10^5^ cells/well and incubated overnight in an incubator (37°C, 5% CO_2_). The test concentration was configured based on the results from keratinocyte toxicity and cytomorphological tests. When the cell confluence reached 40%–60%, the drugs were administered into the cells in each group, which consisted of 4 multiple wells. Test samples with different concentrations were added to the experimental group, whereas a culture solution was added to the control group. All cells were then incubated for 24 h in an incubator (37°C, 5% CO_2_). After incubation the culture solution was removed and the cells were washed and then 1 mL of RNAiso Plus was added to each well. After the cells were dried and lysed, the samples were collected. RNA was extracted and reverse transcribed into cDNA, and then detected by fluorescence quantitative PCR [24]. The primer sequences were as follows: HAS1, forward: 5′‐GCAAGCGCGAGGTCATGTA‐3′, reverse: 5′‐CCAACCTTGTGTCCGAGTCA‐3′; HAS2, forward: 5′‐TCCTGGATCTCATTCCTCAGC‐3′, reverse: 5′‐TGCACTGAACACACCCAAAATA‐3′; HAS3, forward: 5′‐AGCACCTTCTCGTGCATCATGC‐3′, reverse: 5′‐TCCTCCAGGACTCGAAGCATCT‐3′; AQP3, forward: 5′‐CACAGCCGGCATCTTTGCTA‐3′, reverse: 5′‐TGGCCAGCACACACACGATA‐3′.

### 2.5 Immunofluorescence Staining of 3D Epidermis Model

The epidermal model was transferred to a 6‐well plate (0.9 mL of EpiGrowth medium was added). The blank group did not receive any treatment, while the sample group was uniformly distributed in 0.625% sample solution on the model surface. All cells were incubated in an incubator (37°C, 5% CO_2_) for 24 h. After incubation, the surface of the model was rinsed with sterile PBS solution to remove remaining subjects. The model was cut off and fixed with 4% paraformaldehyde for 24 h. 0.5% Triton X‐100 dissolved in phosphate buffer saline was used for section permeabilization for 20 min, and then the sections were blocked with 5% BSA at room temperature for 60 min. Then added 50 μL of different primary antibody solutions (HA, AQP3, Claudin‐1, and FLG) to the corresponding sections and incubated overnight at 4°C. Subsequently, 50 μL fluorescent secondary antibody solution (HA, AQP3, Claudin‐1, and FLG) for 1 h at room temperature. Finally, 50 μL of mounting medium containing the nuclear dye DAPI (4′,6‐diamidino‐2‐phenylindole dihydrochloride) to each section. Images were taken under a microscope and used for observation. The average fluorescence intensity was analyzed by the Image J software using pictures from skin slices [3].

### 2.6 Cell Protection Rate Test

Cells were inoculated in 24‐well plates and incubated in a 5% CO_2_ incubator at 37°C for 24 h. The test samples were prepared by mixing 0.0625% SCE(T), 0.0625% DOP (100 k–500 kDa), and 0.0625% MC. After 24 h of cell growth in the 24‐well plate, the samples were administered into the cells in all groups, with each group consisting of 3 multiple wells. Both the blank control group (BC) and the negative control group (NC) were added with cell culture medium, whereas the sample group was added with cell culture medium containing test samples. The culture was continued for 24 h at 37°C in a 5% CO_2_ incubator. After removing the culture medium (except for the blank control group), the cells were dried for 20 min at medium wind speed in a small ultra clean table. Subsequently, the culture medium was added and incubated at 37°C in a 5% CO_2_ incubator for 24 h. The cell protection rate was determined by MTT assay [25].

Cell protection rate%=Test groupOD/Blank groupOD×100%.

### 2.7 Verification of Human Moisturizing Effect

Twelve healthy volunteers (6 males and 6 females, the average age was 47.9) with dry forearm flexor skin were selected for the test. All volunteers voluntarily participated in the test and have signed an informed consent form. Gels containing 5% DOP (100 k–500 kDa), 5% SCE(T), 5% MC, and gel formula matrix were applied to different parts of the forearm flexion of the volunteers (Table 1). The moisture content of the skin was measured by the Corneometer before (baseline values) and after 1, 2, and 4 h of application. During the testing process, the measurement area (each 3 cm × 3 cm) was marked on the forearm flexion of the volunteers. Then selected 5 different positions were selected in a flower‐like pattern within the testing area for 5 repeated measurements, and the values were averaged and analyzed.

**TABLE 1:** Gel composition.

Materials	Content (%)			
	5% DOP	5% SCE(T)	5% MC	Formula matrix
Water	To 100	To 100	To 100	To 100
Ammonium acryloyldimethyltaurate/VP copolymer	1.00	1.00	1.00	1.00
Sample	5.00	5.00	5.00	—
1,2‐Hexanediol	0.5	0.5	0.5	0.5
Hydroxyacetophenone	0.5	0.5	0.5	0.5

Abbreviations: DOP, *Dendrobium officinale* polysaccharide; MC, moisturizing complex; SCE(T), *S. crispa* extract high temperature and micro‐pressure treatment group.

Volunteer inclusion criteria: The volunteers were 18–60 years old, with dry forearm skin (skin moisture content was 15 C.U. ~ 45 C.U.). These volunteers were healthy during the test, voluntarily participated in the test, and have signed an informed consent form.

Volunteer exclusion criteria: Volunteers with a history of severe systemic diseases, immunodeficiency, allergic diseases, skin diseases, and allergies to commonly used cosmetics; Within one month, the subject has received skin treatment, beauty treatment, and other clinical tests that may affect the results; Individuals who have used hormone or immunotherapy drugs within the past month; Skin features such as damage, inflammation, and scar lumps in the testing area that affect the test results; Individuals who have participated in other clinical trials at the test site within the past month or currently; Women during pregnancy, lactation, and within six months postpartum.

### 2.8 Depth of Canthus Wrinkles and Skin Elasticity Testing

Volunteer inclusion criteria: Twenty volunteers (9 males and 11 females) aged between 25 and 40 years with visible wrinkles or fine lines in the corner of their eyes were selected. These volunteers were healthy during the test, voluntarily participated in the test, and have signed an informed consent form. Volunteer exclusion criteria: Same as in 2.7.

Half‐face control test was used. Testers used moisturizing complex samples and sample matrices on both sides of their face every day. The sample composition is shown in Table 2. After 0, 2, 4, and 6 weeks, the average depth of crow's feet was measured using the canthus images collected by EvaSKIN. The skin elasticity was measured by an MPA580 skin elasticity tester.

**TABLE 2:** Sample composition.

Materials	Moisturizing complex (%)	Sample formula (%)
Coco‐glucoside	1.50	1.50
Glyceryl stearate	0.80	0.80
Cetearyl alcohol	0.30	0.30
Hydrogenated polyisobutene	2.00	2.00
Caprylic/capric triglyceride	3.00	3.00
Dimethicone	3.00	3.00
Water	75.19	80.19
Disodium EDTA	0.05	0.05
Carbomer	0.10	0.10
Glycerine	5.00	5.00
Xanthan gum	0.10	0.10
Dimethiconol	3.00	3.00
Triethanolamine	0.06	0.06
Sample	5.00	—
Fragrance	0.10	0.10
1,2‐Hexanediol	0.50	0.50
Hydroxyacetophenone	0.30	0.30

*Note:* Sample is the moisturizing complex prepared in Section 2.2.3.

### 2.9 Statistical Analysis

Results are presented as mean ± standard deviation. The mean values were calculated based on data from at least three independent replicate experiments. Statistical analysis was carried out using the SPSS 17.0 system. Statistical differences were determined using the t‐test or one‐way analysis of variance (ANOVA) followed by Dunnett's multiple comparison tests, and *p* < 0.05 indicates a statistically significant difference. In the analysis results, * indicates comparison with the control group or baseline, *p* < 0.05; ** indicates comparison with the control group or baseline, *p* < 0.01.

## 3 Results

### 3.1 Effect of Molecular Weight of DOP on Expression of AQP3 Gene

**FIGURE 1:**
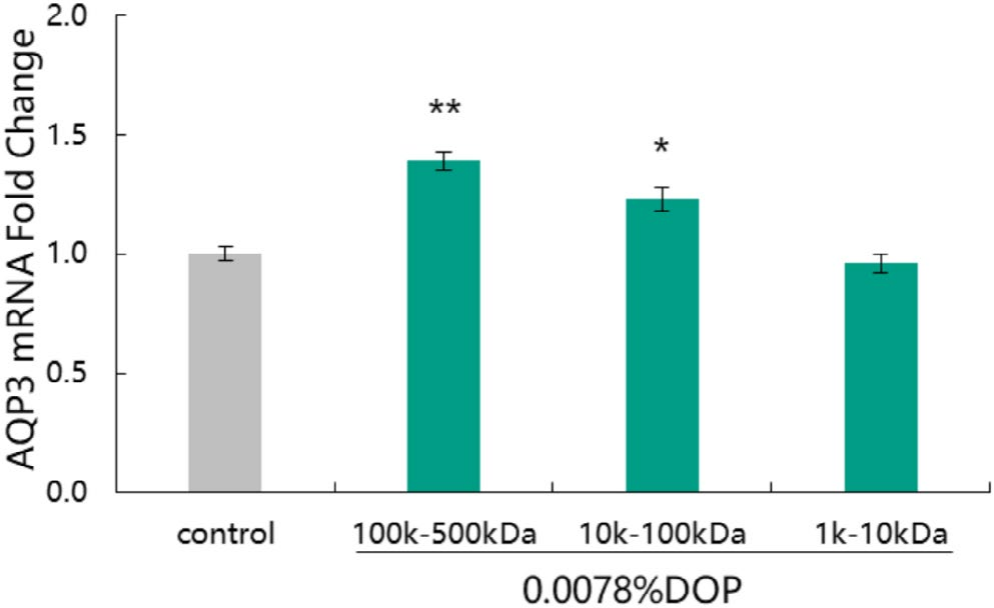
Effect of *Dendrobium officinale* polysaccharide with different molecular weights on the expression of aquaporin 3 gene. *p < 0.05, **p < 0.01 compared with control group. AQP3, aquaporin 3; DOP, *Dendrobium officinale* polysaccharide.

Polysaccharides are the main active components of *D. officinale* , and their molecular weights are closely related to their various biological activities. In this study, the effects of DOP with different molecular weights on the expression of the AQP3 gene were compared, and the results are shown in Figure 1.

Compared to the control group, both DOP (100 k–500 kDa) and DOP (10 k–100 kDa) at a concentration of 0.0078% significantly up‐regulated the expression of AQP3 gene with up‐regulation rates being 39.00% and 23.00%, respectively. DOP (100 k–500 kDa) had a better promoting effect on AQP3 gene expression than DOP with other molecular weights. These results clearly show that polysaccharides with different molecular weights have different effects on the moisturizing gene. Based on the results, we selected DOP (100 k–500 kDa) as the “AQP3 potent factor” and applied it in subsequent moisturizing efficacy experiment.

### 3.2 Analysis of DOP

The polysaccharide content, monosaccharide composition, and polysaccharide bonding structure of DOP (100 k–500 kDa) were analyzed. The results are shown in Tables 3 and 4. The polysaccharide content of DOP was above 90%, indicating that it had high purity. Monosaccharides are the fundamental components of polysaccharides. SóniaS et al. [26] have shown that the immuno‐stimulating activity of polysaccharides is related to their monosaccharides. The quantitative structure–activity relationship models of polysaccharides established by Li et al. [27] shows that polysaccharides with significant antioxidant activity are rich in galacturonic acid, and the antioxidant activity of polysaccharides is influenced by arabinose. The above results indicate that the functional activity of polysaccharides is influenced by both the type and relative content of their monosaccharides. Therefore, it is crucial to study the composition of monosaccharides. As shown in Table 3, DOP mainly included mannose, glucose, galactose, xylose, galacturonic acid, fucose, and arabinose, among which mannose and glucose were the main components. This monosaccharide composition is consistent with that reported in the literature [17].

**TABLE 3:** Polysaccharide content and monosaccharide composition of *Dendrobium officinale* polysaccharide (100 k–500 kDa).

Name	Content (mg/kg)
Guluronic acid	< 1.538
Mannuronic acid	< 1.574
Mannose	728 230.00
Ribose	< 1.295
Rhamnose	< 1.532
Glucuronic acid	< 0.765
Galactose uronic acid	715.00
Glucose	200 125.00
Galactose	6925.00
Xylose	3780.00
Arabinose	645.00
Fucose	715.00
Polysaccharide content	901 200

**TABLE 4:** Analysis of bonding modes of *Dendrobium officinale* polysaccharide (100 k–500 kDa).

Bonding type	Derivative name	MW	Relative molar ratio (%)
T‐Manp	1,5‐Di‐O‐acetyl‐1‐deuterio‐2,3,4,6‐tetra‐O‐methyl‐D‐mannitol	323	0.877
T‐Glcp	1,5‐Di‐O‐acetyl‐1‐deuterio‐2,3,4,6‐tetra‐O‐methyl‐D‐glucitol	323	1.833
4‐Manp	1,4,5‐Tri‐O‐acetyl‐1‐deuterio‐2,3,6‐tri‐O‐methyl‐D‐mannitol	351	71.630
4‐Glcp	1,4,5‐Tri‐O‐acetyl‐1‐deuterio‐2,3,6‐tri‐O‐methyl‐D‐glucitol	351	19.502
3,4‐Manp	1,3,4,5‐Tetra‐O‐acetyl‐1‐deuterio‐2,6‐di‐O‐methyl‐D‐mannitol	379	2.182
4,6‐Manp	1,4,5,6‐Tetra‐O‐acetyl‐1‐deuterio‐2,3‐di‐O‐methyl‐D‐mannitol	379	3.975

Abbreviations: Glcp, glucose furanose; Manp, mannose furanose.

Glycoside bond is the main structural unit of polysaccharide. There is a certain relationship between the types of glycosidic bonds and the functions of polysaccharides. For example, the types of glycosidic bonds have been reported to affect the immune activity of polysaccharides [26]. Therefore, the activity of DOP may be related to the type of its glycosidic bond.

The results from monosaccharide composition analysis showed that the monosaccharide of DOP was mainly neutral sugar, so the bonding types were detected by the neutral sugar methylation method [23]. According to Table 4, the main bonding types of DOP included T‐Manp, T‐Glcp, 4‐Manp, 4‐Glcp, 3,4‐Manp, and 4,6‐Manp, among which 4‐Manp was the main one.

### 3.3 Effect of *S. crispa* Extract on Expression of HAS Gene

**FIGURE 2:**
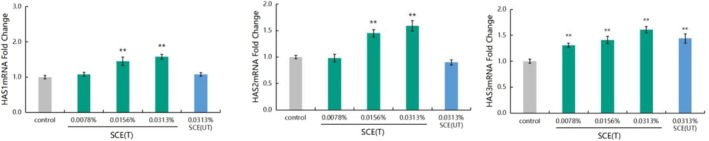
Effect of *Sparassis crispa* extract on hyaluronic acid synthetase gene expression. **p < 0.01 compared with control group. HAS, hyaluronic acid synthetase; SCE(T), *S. crispa* extract high‐temperature and micro‐pressure treatment group; SCE(UT), *S. crispa* extract untreated group.

The effect of SCE(T) on the expression of HAS1, HAS2, and HAS3 genes is shown in Figure 2. Compared to the control group, the 0.0078% SCE(T) could significantly up‐regulate the expression of HAS3 (*p* < 0.01) with an up‐regulation rate of 31%. Additionally, the 0.0156% and 0.0313% SCE(T) samples could significantly up‐regulate the expression of HAS1, HAS2, and HAS3 (*p* < 0.01) with the up‐regulation rates of 45.00%, 45.00%, and 41.00%, respectively for 0.0156% SCE(T) and 58.00%, 59.00%, and 61.00%, respectively for 0.0313% SCE(T).

In addition, the effects of high‐temperature and micro‐pressure treatments on the efficacy of *S. crispa* were compared. We found that the 0.0313% SCE(UT) increased the expression of HAS1, HAS2, and HAS3 genes by 8.00%, −10.00%, and 44.00% respectively, which were lower than those increased by SCE(T) at the same concentration.

The results showed that SCE could promote the expression of HAS1, HAS2, and HAS3 genes, which then promote the synthesis of endogenous hyaluronic acid. They also showed that the effect of SCE was improved to a certain extent after high‐temperature and micro‐pressure treatments.

### 3.4 Effect of Moisturizing Complex on Expression of Moisturizing Proteins

Based on the above results, we screened out DOP with appropriate molecular weights and employed it to promote the expression of AQP3. We then combined it with *S. crispa* , which had the effect of promoting the expression of HAS, to form a moisturizing complex and thereafter investigated its moisturizing effect.

**FIGURE 3:**
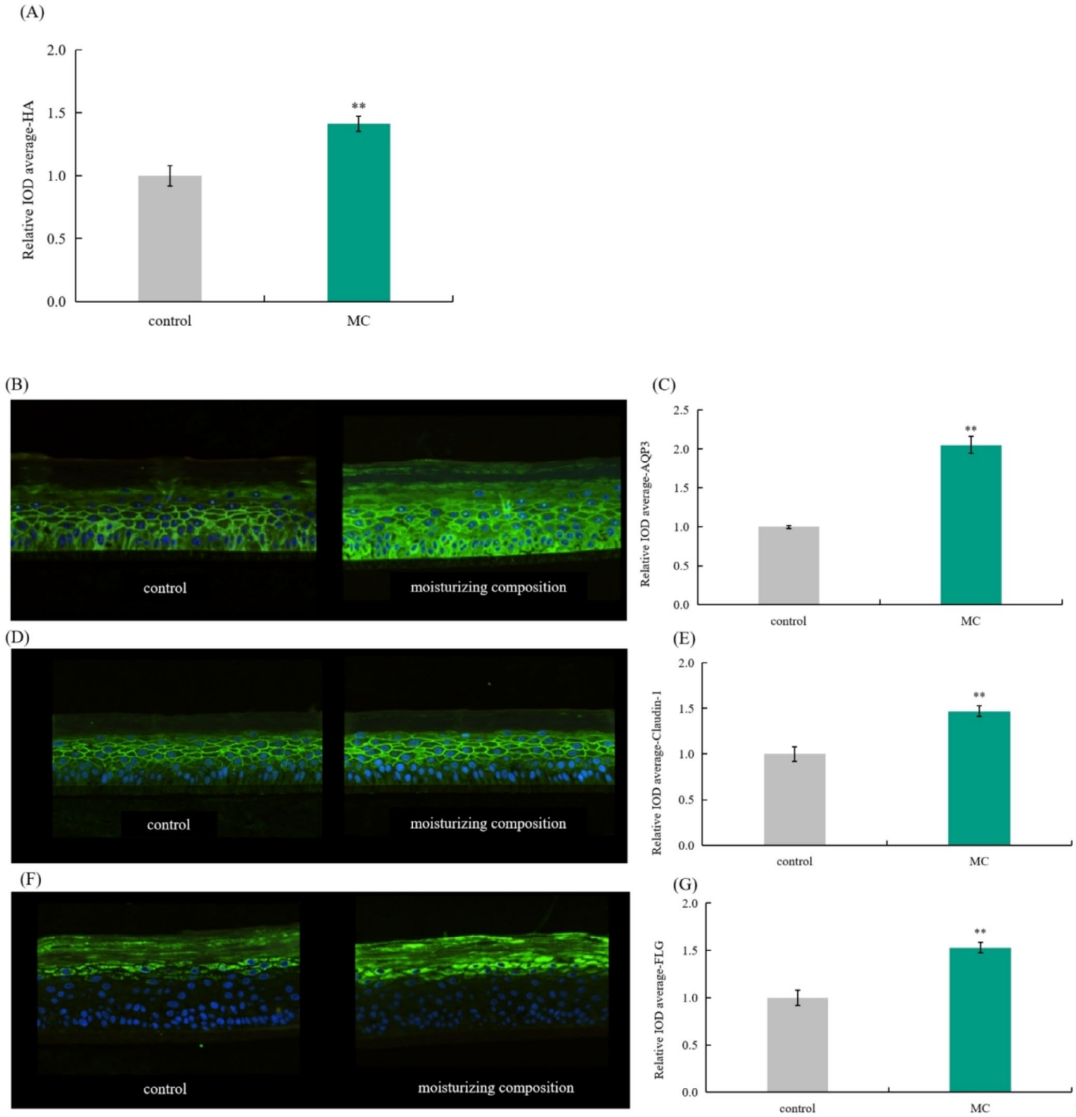
Effect of moisturizing complex on expression of moisturizing‐related proteins. (A) Effect of MC on expression of HA protein; (B, C) Effect of MC on expression of AQP3 protein; (D, E) Effect of MC on expression of Claudin‐1 protein; (F, G) Effect of MC on expression of FLG protein. **p < 0.01 compared with control group. AQP3, aquaporin 3; FLG, filaggrin; HA, hyaluronic acid; IOD, integral optical density; MC, moisturizing complex.

As shown in Figure 3, the expression levels of HA, AQP3, Claudin‐1, and FLG proteins significantly increased after 0.625% MC treatment with rates of 41.00%, 105.00%, 47.00%, and 53.00%, respectively. At the same time, the immunofluorescence map showed that the fluorescence intensity of the epidermal model significantly enhanced after moisturizing complex treatment.

### 3.5 Cell Protection Rate

**FIGURE 4:**
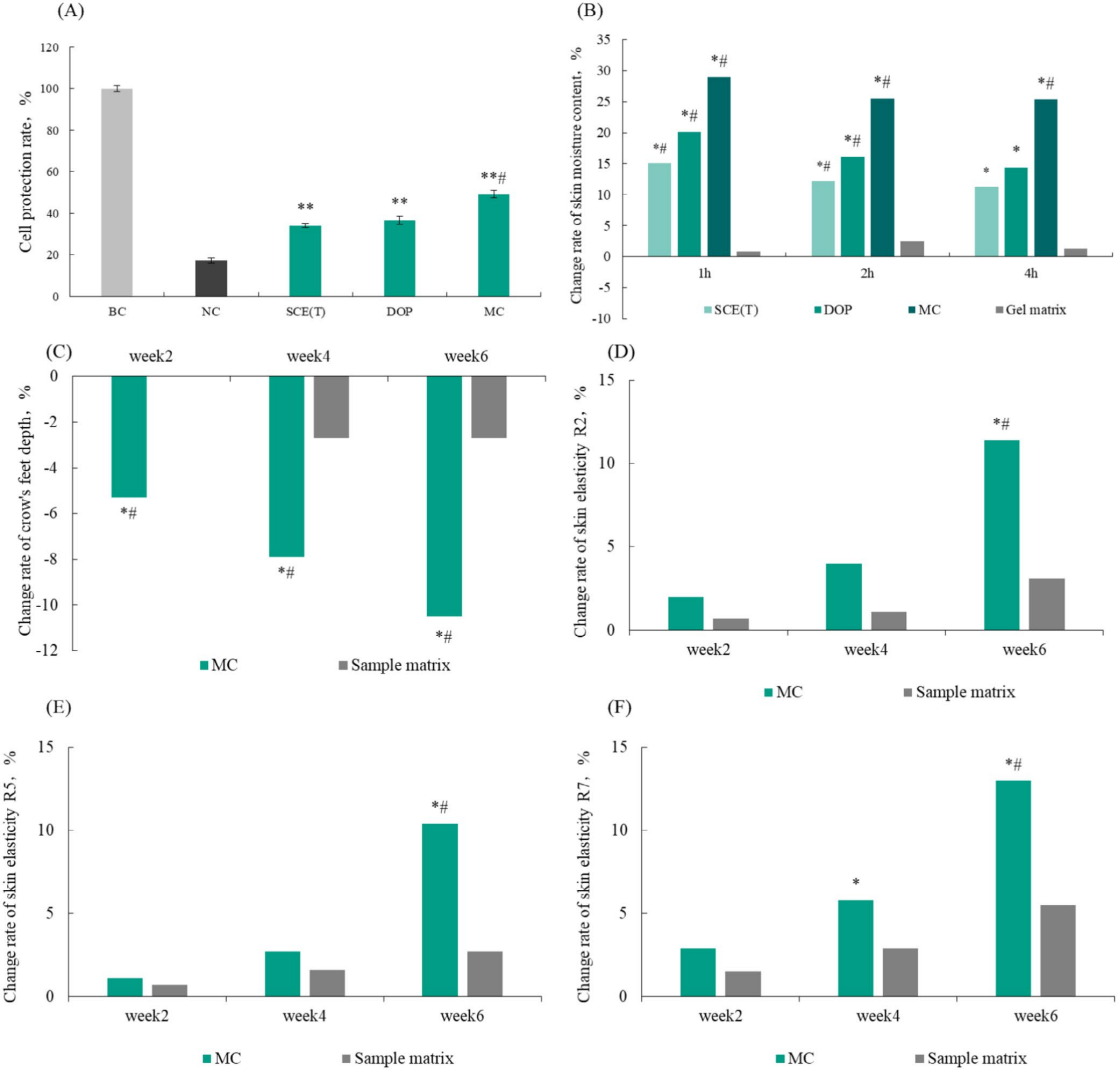
The efficacy of moisturizing complex. (A) cell protection rates of samples in various groups (**p < 0.01 compared with negative control group, #*p* < 0.05 compared with SCE(T) and DOP groups. Change rate was calculated using mean values by: change rate (%) = [(Tx ‐T0)/T0] × 100%); (B) Change rate of skin moisture content (*n* = 12, *p < 0.05 vs. baseline, #*p <* 0.05 vs. gel matrix); (C–F) change rate of crow's feet depth and skin elasticity (*n* = 20, *p < 0.05 vs. baseline, #*p <* 0.05 vs. matrix group). BC, blank control group; DOP, *Dendrobium officinale* polysaccharide; MC, moisturizing complex; NC, negative control group; SCE(T), *S. crispa* extract high temperature and micro‐pressure treatment group.

The ability of SCE(T), DOP, and MC to resist drying injury was tested using a cell drying injury model, and the results are shown in Figure 4A. The cell protection rates of 0.0625% SCE(T) and DOP were 34.12% and 36.71%, respectively. By contrast, the cell protection rate of MC was 49.21%, which was significantly higher than that of the negative control group (damaged as a result of drying) (*p* < 0.001) and was significantly different from that of SCE(T) and DOP. It can be seen that the combination of SCE(T) and DOP could improve the cell protection ability, prevent cell water loss, and maintain the structural integrity of cells, in turn protecting cells from drying injury.

### 3.6 Enhancement of Skin Hydration

Results on change rates of skin moisture content after 1, 2, and 4 h after sample application are shown in Figure 4B. As can be seen, both SCE(T) and DOP were able to increase the skin moisture content, and their combination could enhance the moisturizing effect. The moisturizing effect of the combination was superior to that of each sample alone. Therefore, combining *S. crispa* and DOP led to the synergistic effect, and MC had a good moisturizing effect that resulted in increased skin moisture content.

### 3.7 Improvement of Crow's Feet and Skin Elasticity After the Application of MC

Results on the depth of crow's feet and change rate of skin elasticity after the application of MC are shown in Figure 4C,D–F. After applying with 5% moisturizing composition for 2, 4, and 6 weeks, the crow's feet depth was reduced by 5.3%, 7.9%, and 10.5%, respectively. Additionally, after using the product for 6 weeks, the R2, R5, and values increased by 11.4%, 10.4%, and 13.0%, respectively. The differences were statistically significant (*p* < 0.05) compared with the matrix group.

**FIGURE 5:**
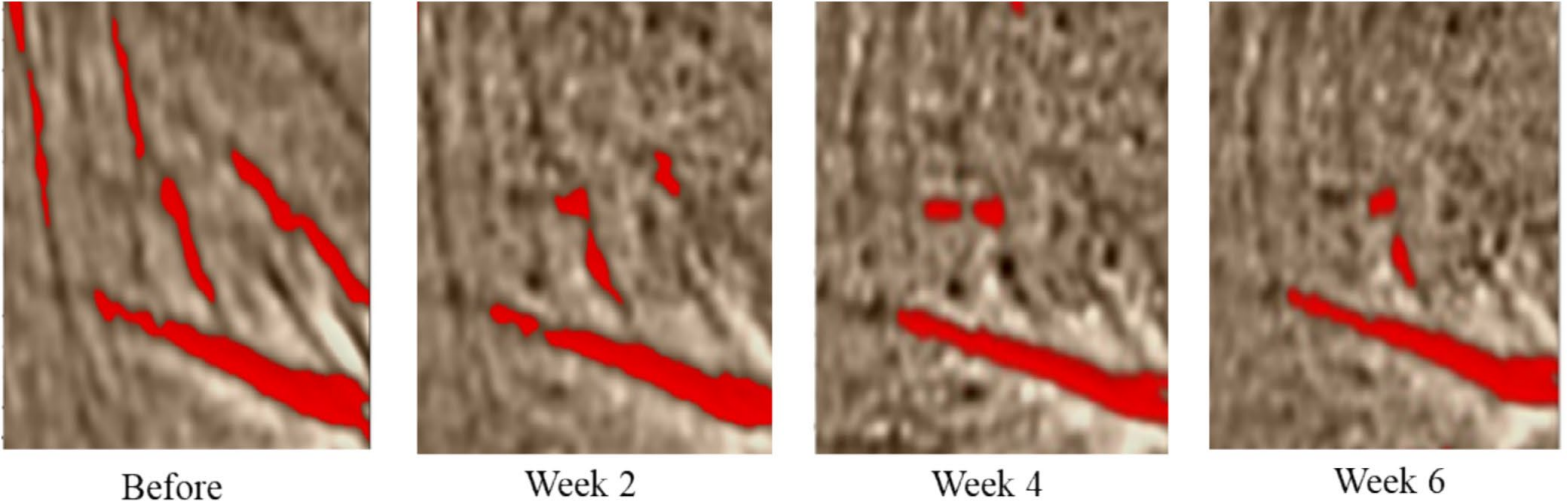
Improvement of crow's feet.

After 0, 2, 4, and 6 weeks, canthus wrinkle images were collected and analyzed by EvaSKIN. The red lines in the picture represent the degree of wrinkles, and the more red lines, the more wrinkles. According to the analysis result of canthus wrinkle image, after using the MC sample for 6 weeks, the corner wrinkles were significantly reduced (Figure 5). These results show that the moisturizing composition can effectively improve crow's feet and enhance the elasticity of facial skin, which is indicative of a certain anti‐aging effect.

## 4 Discussion

In recent years, more and more plant polysaccharides are used in moisturizing cosmetics, and among them is *D. officinale* , which is rich in polysaccharides; its activity is correlated with its polysaccharide content [28, 29]. Due to the high content of polysaccharides in *D. officinale* , our understanding of the structure and activity of polysaccharides has gradually deepened. It has been reported that the biological activities of polysaccharides are often closely related to their chemical structures [16, 17]. Molecular weight is one of the important factors affecting the pharmacological activity of DOP. Studies have shown that low‐molecular weight polysaccharides have higher antioxidant activity than high‐molecular weight polysaccharides [18]. In addition, the correlation between molecular weight and pharmacological activity of polysaccharides has been confirmed in polysaccharides from other plants. For example, one study shows that the greater the molecular weight of longan polysaccharide, the stronger the ability to activate the immune activity of mice [30]. Therefore, it is necessary to study the structure–activity relationship of polysaccharides to better understand and predict their functions.

Based on the above, DOP with different molecular weights were separated, and their effect on the expression of moisturizing genes was explored. Our result showed that at the same concentration, DOP with molecular weights of 100 k–500 kDa was better than that with molecular weights of 10 k–100 kDa and 1 k–10 kDa. Based on this result, DOP (100 k–500 kDa) was selected as the “AQP3 potent factor”. The results also showed that the molecular weight had a certain influence on the expression of moisturizing genes; thus, it is necessary to screen polysaccharides with appropriate molecular weights using the specific efficacy as a criterion in order to better improve the efficacy of the product.

Hyaluronic acid (HA) is a high molecular weight glycosaminoglycan with high hydrophilicity that can promote skin hydration and further affect cell proliferation, inflammation, and wound healing [31]. In cosmetics, HA activity depends on its molecular weight. Hydration is due to high molecular weight HA, and the anti‐aging effect is due to low molecular weight HA. The effect of medium molecular weight HA is represented by cell repairing and wound healing [32].

At present, a humectant hyaluronic acid (HA) is widely used in cosmetics; however, there are some disadvantages in the application process. HA can be degraded into small molecules under the action of hyaluronidase, and this may cause allergies. In vitro injection of HA also has medical limitations. Therefore, by promoting the synthesis of HA in human cells, we could achieve a higher moisturizing effect [33, 34]. HA is synthesized by HAS on the inner surface of the cell membrane and then decomposed into the extracellular matrix. Different subtypes of HAS catalyze the synthesis of HA chains with different lengths; for example, HAS1 and HAS2 catalyze the synthesis of large‐sized HA, whereas HAS3 catalyzes the synthesis of small‐sized HA [35]. This paper studied the effect of *S. crispa* on the expression of the HAS gene. The results showed that 0.0078% SCE(T) could significantly promote the expression of the HAS3 gene (*p* < 0.01), and 0.0156% and 0.0313% SCE(T) could significantly promote the expression of the HAS1, HAS2, and HAS3 genes (*p* < 0.01), in turn promoting the synthesis of endogenous HA.

Based on our findings, both DOP and SCE were considered as potential moisturizing plants. Therefore, we combined them into moisturizing compositions and evaluated their effect on the expression of moisturizing‐related proteins in vitro. We found that compared with that of the control group, the expression levels of HA, AQP3, Claudin‐1, and FLG increased by 41.00%, 105.00%, 47.00%, and 53.00%, respectively, after the application of 0.625% moisturizing composition. Additionally, the fluorescence intensity of cells was significantly enhanced.

AQP3 is the most expressed AQP gene in the human skin; it can promote the transport of water, glycerol, and urea and has moisturizing and repairing effects on skin [15, 36]. Research has demonstrated that compared to wild‐type mice, AQP3 gene‐knockout mice have dry skin and reduced skin elasticity [37]. In addition, the expression level of AQP3 in the skin of old mice is lower than that of young mice, which suggests that AQP3 may be involved in the reduction of water content in the dermis of old mice [38]. Skin AQP3 is involved in the development of many skin diseases, such as eczema, diabetic xeroderma, psoriasis, and hyperplastic dermatosis; thus, it is an important molecule that controls skin function [39].

The water content of skin is as high as 70% in viable epidermis, and this number drops sharply to 15%–30% at the junction between stratum granulosum and stratum corneum. This area is also known as the corneo‐epidermal junction (C.E.J.), which helps to preserve the most important solutes and water in viable epidermis [40]. One study has shown that there are tight junction (TJ) structures at the junction between stratum corneum and stratum granulosum that function to control paracellular permeability [41]. TJ proteins play an important role in skin hydration not only by strengthening the skin barrier but also by acting as a water barrier through regulating the passage of solutes [42]. Claudin‐1 is widely present in normal skin and is the main component of epidermal TJ [43]. Furuse et al. [32] have reported that mice lacking the tight junction protein claudin‐1 can die due to significant body dehydration. It can be seen that the tight junction structures play an important role in the skin barrier. Thus, when the skin barrier function is damaged, moisture in the skin can be easily lost, causing dry skin problems.

Natural moisturizing factors (NMF) in the keratinocytes of the stratum corneum can effectively absorb water and strengthen the water‐locking barrier. NMF is formed by the decomposition of FLG in the epidermis. FLG is an important component of the keratinized envelope that maintains the integrity of the stratum corneum [3]. While FLG localizes in the stratum corneum, its precursor, profilaggrin, localizes in the stratum granulosum. When cells reach the stratum corneum, profilaggrin is rapidly dephosphorylated by Caspase‐14 to form FLG, which is then hydrolyzed into various NMF [44].

Therefore, the moisturizing compound extract can simultaneously promote the expression of HA, AQP3, Claudin‐1, and FLG proteins to regulate water transport and barrier function. As a moisturizing ingredient, it is expected to provide good effects when added to skin care products. The verification of the efficacy of the moisturizing composition consisting of SCE and DOP showed that the moisturizing composition could effectively increase the moisture content of the skin, and the effect is better than the effect of SCE or DOP alone. Finally, the moisturizing compound was proven to improve crow's feet and skin elasticity, which could be used as scientific support for its future application in cosmetics.

## 5 Conclusions

In summary, the effects of DOP with different molecular weights and SCE on the expression of moisturizing genes were studied. The results showed that 100 k–500 kDa DOP could better promote the expression of the AQP3 gene than 10 k–100 kDa and 1 k–10 kDa. Moreover, SCE could effectively promote the expression of HAS1, HAS2, and HAS3 genes and the endogenous synthesis of HA. Further verification of the efficacy of the moisturizing compound revealed that it could effectively improve the expression of HA, AQP3, Claudin‐1, and FLG proteins and prevent drying injury. When applied to the human body, it could effectively increase skin moisture content and improve crow's feet and skin elasticity. Taken together, *D. officinale* and *S. crispa* had good moisturizing effects, and as natural plant humectants, they may have broad applications in future moisturizing cosmetics.

## Author Contributions

Taiju Di drafted the work and interpreted of data; Hong Meng and Hankun Ren designed the work; Peina Zha and Weihong Zhang substantively revised it; Yueheng Liu worked on conceptualization, project administration and supervision. All authors read and approved the final manuscript.

## Ethics Statement

Before the study, benefits and potential risks were explained to the subjects, and the informed written consent of the participants was obtained, and each of the participants signed the informed consent.

## Conflicts of Interest

The authors declare no conflicts of interest.

## Data Availability

The data that support the findings of this study are available from the corresponding author upon reasonable request.
